# Clinical Immunological Correlations in Patients with Multiple Sclerosis Treated with Natalizumab

**DOI:** 10.3390/brainsci10110802

**Published:** 2020-10-30

**Authors:** Smaranda Maier, Mihaela Simu, Adina Hutanu, Laura Barcutean, Septimiu Voidazan, Zoltan Bajko, Anca Motataianu, Irina Lata, Rodica Balasa

**Affiliations:** 1 Neurology 1 Clinic, Emergency Clinical County Hospital Mures, 540136 Targu Mures, Romania; maier_smaranda@yahoo.com (S.M.); laurabarcutean@gmail.com (L.B.); bzoltan2003@yahoo.com (Z.B.); motataianuanca@gmail.com (A.M.); rodica.balasa@umfst.ro (R.B.); 2 Neurology Department, University of Medicine, Pharmacy, Science and Technology, “G. E. Palade”, 540139 Targu Mures, Romania; 3 Department of Neurology, University of Medicine and Pharmacy “Victor Babes”, 300041 Timisoara, Romania; mihaelasimu6713@gmail.com; 4 Neurology Clinic, County Emergency Clinical Hospital “Pius Brinzeu”, 300732 Timisoara, Romania; irinalata@yahoo.com; 5 Laboratory Medicine Department, University of Medicine, Pharmacy, Science and Technology, “G. E. Palade”, 540139 Targu Mures, Romania; 6 Immunology Laboratory, Center for Advanced Medical and Pharmaceutical Research, George Emil Palade University of Medicine, Pharmacy, Science and Technology, “G.E. Palade”, 540139 Targu Mures, Romania; 7 Epidemiology Department, University of Medicine, Pharmacy, Science and Technology, “G. E. Palade”, 540139 Targu Mures, Romania; septi_26_07@yahoo.com

**Keywords:** multiple sclerosis, natalizumab, mechanism of action, pro and anti-inflammatory cytokines

## Abstract

Natalizumab (NAT) was the first disease modifying therapy used for the treatment of relapsing-remitting multiple sclerosis (MS) that was designed with a specific mechanism of action that targets an important step of the MS immunopathology, directly blocking the T lymphocyte intrusion in the central nervous system. Initially, it was considered that NAT carried no biological effects on the peripheral immune response. The purpose of our study was to assess the effects of NAT on the peripheral pro and anti-inflammatory cytokines and to reveal possible correlations between them and the clinical activity of the disease. We noticed a significant decrease in interleukin (IL)-17, tumor necrosis factor-alpha (TNF-α) and IL-31 serum levels in treated patients. The lack of relapses during the study was associated with low baseline IL-17 level. The patients that had an increase in the disability score during the study had significantly lower IL-17 and higher IL-1β baseline levels. IL-17 can be used as a biomarker for disease activity but also for progression assessment in NAT treated patients. NAT has a far more complex mechanism compared to what was initially believed, besides modulating lymphocyte trafficking through the blood–brain barrier, it also changes the peripheral levels of pro and anti-inflammatory cytokines in MS patients.

## 1. Introduction

Multiple sclerosis (MS), the most common disabling pathology of the central nervous system (CNS) in young adults, and is characterized by two entwining pathophysiological processes: an autoimmune inflammatory response, mediated by auto-reactive lymphocytes, and a neurodegenerative process which mediates myelin sheath destruction both in the brain and the spinal cord [1].

Concerning MS pathology, there is clear evidence that an autoimmune adaptive inflammatory response, which involves T and B lymphocytes directed against various CNS molecules, exists [2,3]. Steinman and his team of researchers from Stanford concentrated their research on the molecules that actively guide the migration of T lymphocytes from the periphery towards the blood–brain barrier (BBB), a phenomenon known as “zip code hypothesis”. Subsequently, Steinman and colleagues assessed a number of monoclonal antibodies directed against adhesion molecules and noted that the antibodies against α4 integrin, an essential protein necessary for lymphocyte adherence to the BBB, prevent the intrusion of inflammatory cells in the brain parenchyma [2,4]. After more than a decade since the discovery of this molecule, in 2004, natalizumab (NAT), a humanized antibody directed against α4 integrin, was approved by the Food and Drug Administration for the treatment of relapsing-remitting MS (RRMS) [5,6].

NAT was the first disease modifying therapy (DMT) used for the treatment of RRMS, designed with a specific mechanism of action (MoA) which targets an essential step of the MS immunopathology—lymphocyte intrusion in the CNS, thus preventing acute demyelinating episodes [7]. The efficacy of NAT was endorsed by numerous clinical studies. The AFFIRM study demonstrated that over 2 years of NAT treatment significantly reduced new T2 lesions, active lesions, clinical relapses, and the progression of disability as compared to placebo [8].

The objectives of this study are the following: (1) assessment of peripheral pro- and anti-inflammatory cytokines production under the treatment with NAT, especially the Th17 lineage, due to the important role this subtype of lymphocytes plays in the pathogenesis of MS, and (2) assessment of various correlations between serum cytokines induced by NAT treatment and the disease activity during the treatment period.

## 2. Materials and Methods

We performed a prospective study that included 60 RRMS patients from two Regional MS centers in Romania. A total of 31 patients were actively followed and treated in the MS Center of Timisoara and 29 patients were actively followed and treated in the MS center of Targu Mures. The study took place between 1 January 2019 and 31 December 2019. The study included 33 healthy controls (HC), both age- and sex-matched to the MS subjects. All the patients included in the study were either actively treated with NAT or NAT treatment was started at the beginning of the study. NAT was administered by intravenous infusion at a dose of 300 mg at 4-week intervals. The inclusion criteria were: (1) RRMS diagnosis according to the 2017 McDonald criteria [9], (2) patients were actively treated with NAT or eligible for NAT treatment, (3) over 18 years old, and (4) no relapses or corticosteroid treatment administered one month prior to sample assessment. The exclusion criteria were: (1) contraindications for NAT treatment administration, (2) systemic infection, and (3) neoplastic disorders. All MS patients and HC signed the informed consent. The study was conducted in accordance with the declaration of Helsinki.

Demographical data were collected for both MS patients and HC. The assessment of MS patients included clinical parameters such as disease duration, duration of NAT treatment, number of relapses before and during the study, neurological examination at the beginning and at the end of the study, and Expanded Disability Status Scale (EDSS) calculation.

Blood samples were taken from both MS patients and HC at the beginning of the study and at the end of the study (after a mean interval of 6.96 ± 1.28 months) from MS patients only. The venous samples were harvested in the morning in a clot accelerator tube from subjects in a fasting state, before administering the NAT infusion. Tubes were left for 20 min at room temperature for clot formation and were then centrifuged at 3500 rotations per minute. The subsequent serum was aliquoted and stored at −70 °C until assessment. A premixed panel, Bio-plex Pro Human Th17 Cytokine Panel (Bio-Rad Laboratories, Inc., Hercules, CA, USA), tested on a Flexmap 3D Analyzer, was used to evaluate the serum level of the following 15 cytokines: interleukin (IL)-1β, IL-4, IL-6, IL-10, IL-17A, IL-17F, IL-21, IL-22, IL-23, IL-25, IL-31, IL-33, interferon-gamma (IFN-γ), sCD40L, tumor necrosis factor-alpha (TNF-α).

We obtained detectable serum levels for 9 out of the 15 cytokines included in the panel. Cytokines detected at the beginning of the study were noted as: IL-1β_0, IL-10_0, IL-17_0, IL-21_0 IL-23_0, IL-31_0, IL-33_0, sCD40L_0, TNF-α_0, and at the end of the study were noted as: IL-1β_1, IL-10_1, IL-17_1, IL-21_1, IL-23_1, IL-31_1, IL-33_1, sCD40L_1, TNF-α_1, in order to facilitate data presentation.

For statistical calculations, Statistical Package for Social Sciences (SPSS, version 22, Chicago, IL, USA) was used. Data were considered as nominal or quantitative variables. Nominal variables were described as absolutes and relative frequencies (%) and the association between them was analyzed by Pearson’s Chi-square test or Fisher’s Exact Test. Quantitative variables were tested for normality of distribution using the Kolmogorov–Smirnov test and were characterized by median and percentiles (25–75%) or by mean and standard deviation (SD), when appropriate. Quantitative variables were compared using the Wilcoxon and Mann–Whitney test. The correlation between quantitative variables was assessed using Pearson correlation or Spearman’s rho, when appropriate. We interpreted all tests against a *p* = 0.05 significance threshold and statistical significance was considered for *p*-values below the significance threshold.

## 3. Results

The study included 60 RRMS patients and 33 HC. Regarding the gender distribution, 39(65%) female and 21(35%) male MS patients were included, and in the HC group, 22(66.6%) were female and 11(33.3%) were male. The mean age of MS patients at study inclusion was 38.1 ± 7.92 years, and the mean age of HC at inclusion was 37.8 ± 6.4 years. Demographic data for MS patients and HC, as well as disease-related data for MS patients, can be found in Table 1.

Serum values of the studied cytokines were compared in MS patients at the beginning and end of the study using the Wilcoxon nonparametric test. A statistically significant decrease in serum IL-17 (*p* = 0.031), TNF-α (*p* = 0.022), and IL-31 (*p* ˂ 0.001) was observed at the end of the study.

The Mann–Whitney test was used to compare serum cytokine values in MS patients at the beginning of the study with those of HC. Patients with MS had significantly higher serum values of IL-23 (*p* = 0.05) and IL-31 (*p* = 0.021) compared to HC.

**Figure 1:**
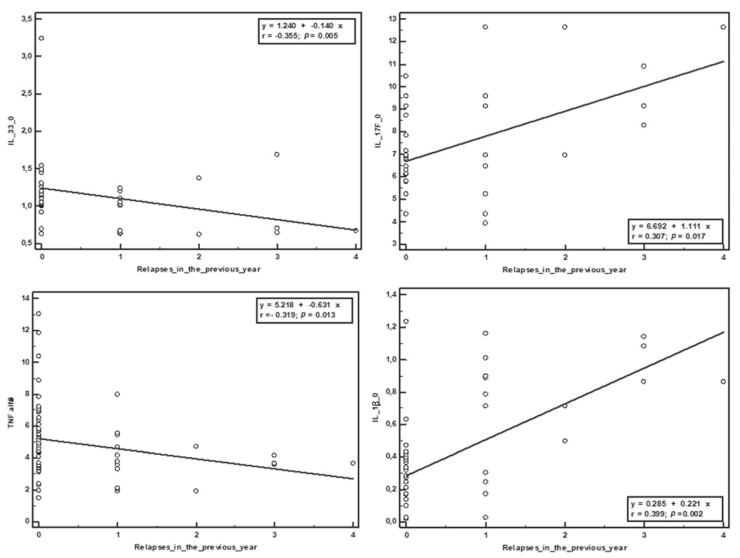
Correlations between the relapses in the year prior to the study and the initial serum cytokine levels.

**Figure 2:**
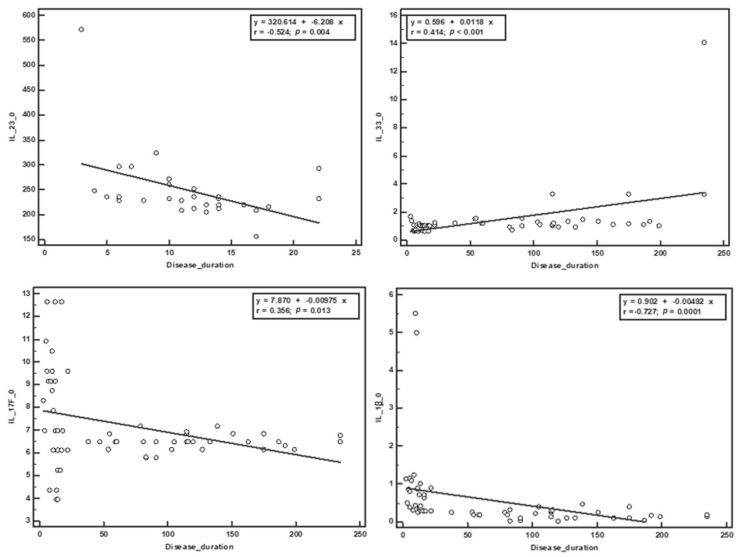
Correlations between the disease duration and the initial serum cytokine levels.

The Spearman correlation was used to establish the relationship between the serum values of the cytokines obtained at the beginning of the study and the clinical parameters. No significant correlation was observed with EDSS score at the beginning and end of the study. Negative correlations were obtained between the duration of treatment and IL-23 (r = −0.405, *p* = 0.029) and IL-1β (r = −0.363, *p* = 0.004), and between the duration of the disease and IL-23 (r = −0.524, *p* = 0.004), IL-17 (r = −0.356, *p* = 0.005), IL-1β (r = −0.727, *p* ˂ 0.001) and IL-31 (r = −0.348, *p* = 0.006). The duration of the disease was positively correlated with IL-33 (r = 0.414, *p* = 0.001). The number of relapses from the year before the study was negatively correlated with sCD40L (r = −0.279, *p* = 0.031), IL-33 (r = −0.355, *p* = 0.005), and TNF-α (r = −0.319, *p* = 0.013), and positively correlated with IL-17 (r = 0.307, *p* = 0.017) and IL-1β (r = 0.399, *p* = 0.002). The number of relapses in the study period positively correlated with the initial serum values of IL-17 (r = 0.289, *p* = 0.025) and negatively correlated with IL-33 (r = −0.263, *p* = 0.042). (Table 2, Figure 1 and Figure 2).

Depending on the presence or absence of relapses during the study, patients were divided into two groups, and the initial and final values of the cytokines were compared between the two groups. It was observed that patients who did not have recurrences during the study had significantly lower initial serum IL-17 values compared to those who experienced recurrences during the study (*p* = 0.05).

Depending on the evolution of the EDSS score during the study, patients were divided into three groups: (1) Stationary EDSS, (2) Decreased EDSS, and (3) Increased EDSS. Serum cytokine values were compared between the three groups and the following observations were noted:

- IL-33: Patients in whom the EDSS score increased had significantly lower initial and final serum values compared to the other two groups.
- IL-17: Patients in whom the EDSS score increased during the study had significantly lower baseline serum values compared to patients in whom the EDSS score decreased (*p* = 0.01).
- IL-1β: Patients in whom the EDSS score increased during the study had statistically significantly higher initial serum values compared to those in whom the score decreased or remained stationary (*p* = 0.024).

The initial values were compared with the final values in the three groups of patients and the following was observed (Table 3):

- A statistically significant decrease in IL-17 values in those for whom the EDSS score decreased.
- A statistically significant decrease in TNF-α in those for whom the EDSS score remained stationary.
- A statistically significant decrease in IL-31 in those whose EDSS score was stationary or decreased.

Depending on the duration of treatment with NAT, three groups were selected: (1) naïve, (2) 1–12 months of treatment, and (3) 13–24 months of treatment. Serum cytokine levels were compared between these groups at the beginning of the study and the following observations were noted: IL-1β and IL-31 serum values statistically significantly decreased, relevant to the duration of the treatment for the three groups.

To understand the effect of NAT treatment on serum levels of pro- and anti-inflammatory cytokines, baseline and final cytokine values were compared in the naïve patient group and a statistically significant decrease in IL-17, IL-33, and IL-31 was observed after approximately 6 months of treatment with Natalizumab. (Table 4)

## 4. Discussion

NAT is a specific, ingeniously-crafted DMT with a well-established MoA. By blocking vascular cell adhesion molecule 1 (VCAM-1) at the level of the endothelial cells, NAT inhibits lymphocyte trafficking through the BBB into the cerebral parenchyma, thereby reducing inflammation and preventing the genesis of demyelinating plaques. Initially, it was considered that NAT carried no other biological effects beyond directly inhibiting lymphocyte passage [7]. This MoA, especially with an anti-inflammatory effect, may be combined in the future with molecules that stimulate remyelination, such as with the anti-LINGO-1 antibody [10,11].

For reasons that are still uncertain, studies have demonstrated that prolonged NAT treatment, over a period of 6 months, is associated with an increase in T CD4+ and CD8+ pro-inflammatory cells in the periphery, which may explain the rebound activity after cessation [12,13]. Benkert et al. revealed that NAT carries an upregulating effect on Th17 cytokines and inhibits IL-10. The same group of researchers noticed that the administration of NAT leads to an increase in IL-17 cells in patients with an already increased baseline level. Regarding the Th1 lineage, if the baseline levels of Th1 cells were increased before NAT administration, Th1 cells decreased after treatment, and conversely, if the baseline levels of Th1 cells were decreased prior to treatment, NAT treatment led to an increase in Th1 cells in the periphery. This study demonstrated that, in addition to blocking activated lymphocyte migration throughout the BBB, NAT acts as a direct co-stimulator of the T cell population, mildly shifting the balance towards a pro-inflammatory phenotype in the periphery [14].

To assess the changes induced by NAT in the periphery, we focused on IL-17, a cytokine produced by Th17 cells. The Th17 subtype has strong pro-inflammatory properties that play an essential role in MS immunopathogenesis, both in initiating and maintaining the progression of the immune response [15,16,17].

Another study reported that, 6 months after NAT treatment was started, patients presented with increased levels of IFN-γ and TNF-α, reinforcing the fact that the complex MoA of NAT is not fully understood, despite being specifically created to act solely as an inhibitor for activated T lymphocytes at the level of the BBB [18]. A plausible theory might be that by inhibiting activated T lymphocyte trafficking at the level of the BBB, they are sequestered in the peripheral circulation or, through unknown causes, NAT leads either directly or indirectly to T lymphocyte activation [12]. One study performed by Buhler at al. could explain this theory, as it demonstrated that the number of T CD4+ cells that secrete IL-17 in the periphery is significantly higher in NAT-treated patients compared to untreated MS patients or HC [19].

By actively following NAT-treated patients over a period of 12 months, Kivisäkk et al. noticed an increase in IL-17-secreting CCR6+ cells and IFN-γ-secreting CCR5+ cells, along with a decrease in Th2 cytokines, which reflects an increased Th1/Th2 ratio in the peripheral blood [12]. Th17 lymphocytes can penetrate the CNS using the CCR6–CCL20 axis because they express the chemokine receptor CCR6+ that permits BBB passage at the level of the choroid plexus, where endothelial cells are abundant in CCL20. This might be a possible explanation as to why some patients continue to experience relapses while undergoing NAT treatment [17].

In the present study, NAT treatment induced a significant decrease in serum IL-17 in the naïve patient group. Similar observations were obtained by our team in an anterior study, and other international researchers have reported the same findings [20,21]. One explanation for these results may be that the above-mentioned studies were based on cell differentiation tests of peripheral Th lymphocytes, while our study focused on protein determinations. Another explanation might be secondary to the genetic variability of the studied populations. Mameli et al. demonstrated that MS patients exhibit a high expression of IL-17, IFN-γ, TNF-α, and IL-6 compared to HC. After 6 months of NAT treatment, a significant decrease was reported in the genic expression for IL-17a, IFN-γ, and TNF-α, and microRNA analysis revealed a significant decrease in miR-155 and miR-26A. The researchers also determined that MS patients that have lower miR-155 and miR-26A levels after 6 months of treatment simultaneously present lower IL-17 and TNF-α levels, but no impact upon IFN-γ and IL-6 secretion was noted. This study reinforces that Th1 or Th17 immune response in MS patients is dependent on certain microRNA expression before and after NAT treatment [1].

Another potential explanation for the contradictory results regarding the peripheral immune panel of MS patients undergoing NAT treatment is the timing of serum sample assessment. Ramos-Cejudo et al. evaluated the NAT effect upon pro- and anti-inflammatory cytokines at 2 h, 4, 7, and 15 days, respectively 3–5, 9–12, and 15–20 months from initial NAT administration. They noted that in the initial stages, NAT induces an increase in both pro- and anti-inflammatory cytokines (IL-4, IL-5, IL-10, IFN-γ, IL-12), but serum levels returned to baseline level after approximately 2 months of NAT treatment. Some pro-inflammatory cytokines (IL-1β, IL-2 and IL-17) had high serum values only after prolonged NAT treatment. TNF-α levels were constant throughout the study [13]. In our cohort, the mean treatment duration was approximately 32 months and only 11 patients were classified as naïve, which might explain the contradictory results.

In our study, the initial elevated IL-17 levels were associated with persistent disease activity (relapses) during the study. The patients that presented relapses during the observation period had both initial and final IL-17 values that were increased compared to patients that were relapse-free. The patients that presented with an increase in EDSS score had lower IL-17 levels compared to the patients that presented with a decrease in EDSS score during the study. In the first group of patients, in which the EDSS score increased, a decrease in IL-17 levels was observed in the 6 months of study. IL-17 positively correlated with the relapses, thus underlying the important role of IL-17 in the pathophysiology of clinically active MS. IL-17 negatively correlated with the progression of disability as calculated by the EDSS. A decrease in IL-17 serum levels may be a biomarker of progression for NAT-treated patients. Regarding the use of IL-17 as a possible biomarker for rebound risk, the data is contradictory. One study reported a fulminating rebound 2 months after NAT cessation in a patient who had a high number of T CD4+ IL-17-producing cells [19], while another study reported low Th17 circulating cells in patients that presented with relapses after NAT cessation, compared with clinically stable patients [22].

As demonstrated in the present study, NAT treatment determined a significant decrease in TNA-α, which contradicts the results of a previous mRNA study [18]. A decrease in TNF-α levels was associated with a lack of disease activity (no relapses, no EDSS progression). Results similar to ours have been reported in the literature [23]. NAT therapy influences the peripheral immune mechanism; it increases the T cell population responsible for pro-inflammatory cytokine secretion, yet also decreases the serum levels of various pro-inflammatory cytokines.

IL-1β is a potent pro-inflammatory cytokine that plays a significant role in the pathogenesis of experimental autoimmune encephalomyelitis (EAE). Numerous studies have demonstrated that IL-1β levels are correlated with the clinical evolution of the disease in murine models and that IL-1β-deficient mice did not develop EAE [24,25,26]. The role of IL-1β in the pathogenesis of MS is not fully understood, but it appears to be actively involved in naïve T cell differentiation towards a Th17 lineage [17]. In our study, an increased baseline IL-1β level correlated with an unfavorable clinical evolution (relapses one year prior to the study; an increase in EDSS during the study). NAT treatment significantly decreased the serum levels of IL-1β, with naïve patients having significantly higher levels compared to patients that were already undergoing NAT treatment for 1–2 years. These results suggest that treatment with NAT influences the peripheral immune panel by significantly decreasing pro-inflammatory cytokines.

IL-33, also known as an “alarmin”, acts as a sentinel whose role is to announce to the host when a pathogen is detected. It carries a dual function dependent on location. If IL-33 is expressed at the level of the nucleus, it will determine remodelation and tissue repair; if it is expressed extracellularly, it induces inflammation. Experimental studies have demonstrated that IL-33 administration reduces the severity of EAE and that, in traumatic spinal lesions, it increases myelin regeneration in affected regions. Most likely by shifting the immune response from pro-inflammatory (mediated by Th1 and Th17) to anti-inflammatory (Th2-dependent), IL-33 carries certain protective effects [27,28,29,30,31,32,33,34]. These hypotheses are supported by the results of our study, which showed that increased IL-33 serum levels were associated with a low number of relapses both one year prior to and during the study and that the patients with an increased EDSS score had significantly lower levels of IL-33 compared to patients that were clinically stable or improved during the treatment.

## 5. Conclusions

The results of our study reinforce that the MoA of NAT is not as simple nor clear as it was intended when the molecule was created. When examining serum levels of pro-inflammatory cytokines in MS patients treated with NAT, we observed a significant decrease in serum levels of IL-17 and TNF-alpha. Low levels of pro-inflammatory cytokines (IL-17, TNF-α, IL-1β) under NAT treatment were associated with favorable clinical outcomes (lack of recurrences, low EDSS score). IL-17 can be used both as a biomarker for disease activity and disease progression in NAT-treated patients.

In order to completely understand this complex MoA, further studies involving genetic testing, cellular cultures, and advanced cellular differentiation, along with protein assessment, are in order.

## Figures and Tables

**Table 1:** Demographic and disease-related data for multiple sclerosis (MS) patients and healthy controls (HC).

	MS Patients	HC
	*n* = 60	*n* = 33
Age	38.1 ± 7.92	37.8 ± 6.4
Sex F/M	39/21	22/11
Disease duration (months)	67.61 ± 67.0	
Treatment duration (months)	32.48 ± 26.58	
Study duration (months)	6.96 ± 1.28	
Number of relapses in the last year	0.46 ± 0.91	
Number of relapses during the study	0.08 ± 0.27	
EDSS at study onset	3.81 ± 1.54	
EDSS at the end of the study	3.76 ± 1.63	

**Table 2:** Correlations between serum cytokine values and clinical parameters. (Spearman correlation).

Variable	Spearman’s Rho	IL-21_0	sCD40L_0	IL-23_0	IL-33_0	IL-17F_0	TNF-α_0	IL-10_0	IL-1β_0	IL-31_0
EDSS at study onset	r	0.129	−0.156	0.237	−0.159	0.058	0.032	−0.055	0.055	0.088
	*p* value	0.326	0.235	0.215	0.226	0.659	0.806	0.674	0.678	0.504
EDSS at the end of the study	r	0.108	−0.099	0.224	−0.089	−0.051	0.046	−0.040	−0.001	0.030
	*p* value	0.414	0.451	0.243	0.500	0.700	0.727	0.759	0.997	0.822
Treatment duration	r	0.021	0.112	−0.405 *	0.236	−0.239	0.080	0.105	−0.363 **	−0.165
	*p* value	0.871	0.393	0.029	0.069	0.065	0.541	0.423	0.004	0.209
Disease duration	r	0.117	0.030	−0.524 **	0.414 **	−0.356 **	0.052	0.001	−0.727 **	−0.348 **
	*p* value	0.375	0.820	0.004	0.001	0.005	0.691	0.994	0.0001	0.006
Relapses in the previous year	r	−0.047	−0.279 *	0.272	−0.355 **	0.307 *	−0.319 *	−0.054	0.399 **	0.232
	*p* value	0.721	0.031	0.153	0.005	0.017	0.013	0.685	0.002	0.074
Relapses during the study	r	−0.002	−0.113	−0.060	−0.263 *	0.289 *	−0.146	−0.094	0.094	0.089
	*p* value	0.989	0.389	0.757	0.042	0.025	0.265	0.474	0.474	0.500

**. Correlation is significant at the 0.01 level (2-tailed). *. Correlation is significant at the 0.05 level (2-tailed).

**Table 3:** Comparison between the initial and final cytokine values according to the Expanded Disability Status Scale (EDSS) score (Wilcoxon’s test).

	IL-21_1 - IL-21_0	sCD40L_1 - sCD40L_0	IL-23_1 - IL-23_0	IL-33_1 - IL-33_0	IL-17F_1 - IL-17F_0	TNF-α-1 - TNF-α_0	IL-10_1 - IL-10_0	IL-1β_1 - IL-1β_0	IL-31_1 - IL-31_0
Z EDSS stationary	−0.385	−0.122	−0.808	−0.389	−1.511	−2.090	−1.646	−1.457	−3.605
*p* value	0.700	0.903	0.419	0.697	0.131	0.037	0.100	0.145	0.0001
Z EDSS decreases	−1.163	−0.314	−1.761	−1.054	−2.032	−1.363	-.135	-.420	−2.207
*p* value	0.245	0.753	0.078	0.292	0.042	0.173	0.892	0.674	0.027
Z EDSS increases	−0.272	−0.535	−1.604	−0.447	−1.000	−0.535	−0.535	−1.069	−1.604
*p* value	0.785	0.593	0.109	0.655	0.317	0.593	0.593	0.285	0.109

**Table 4:** Comparison between the initial and final cytokine values in the naïve patient group (Wilcoxon’s test).

	IL-21_1 - IL-21_0	sCD40L_1 - sCD40L_0	IL-23_1 - IL-23_0	IL-33_1 - IL-33_0	IL-17F_1 - IL-17F_0	TNF-α-1 - TNF-α_0	IL-10_1 - IL-10_0	IL-1β_1 - IL-1β_0	IL-31_1 - IL-31_0
Z	0.0001	−0.663	−0.837	−2.035	−2.023	−0.153	−0.538	−0.869	−2.803
*p* Value	1.000	0.508	0.402	0.042	0.043	0.878	0.590	0.385	0.005
